# Treatment of Asymptomatic Bacteriuria after Kidney Transplantation: A Systematic Review and Meta-Analysis of Randomized Controlled Trials

**DOI:** 10.3390/medicina59091600

**Published:** 2023-09-05

**Authors:** Zhengsheng Rao, Zhiling Wang, Ming Tang, Linguo Shen, Keqin Zhang

**Affiliations:** Department of Urinary Nephropathy Center, Second Affiliated Hospital of Chongqing Medical University, Chongqing 400000, China; rao_zhengsheng@163.com (Z.R.); zhiling718@163.com (Z.W.); tangm1008@cqmu.edu.cn (M.T.); 15025404090@sina.cn (L.S.)

**Keywords:** kidney transplantation, asymptomatic bacteriuria, antibiotic, urinary tract infection

## Abstract

*Background and Objectives:* Asymptomatic bacteriuria (ASB) is prevalent in kidney transplant recipients (KTRs) and is hypothesized to heighten the risk of subsequent urinary tract infections (UTIs). Whether antibiotic treatment of ASB in KTRs is beneficial has not been elucidated. *Materials and Methods:* We carried out a systematic review and meta-analysis of all randomized controlled trials (RCTs) and quasi-RCTs that examined the merits of managing asymptomatic bacteriuria in KTRs. The primary outcomes were rates of symptomatic urinary tract infections (UTIs) and antimicrobial resistance. *Results*: Five studies encompassing 566 patients were included. No significant difference in symptomatic UTI rates was found between antibiotics and no treatment groups (relative risk (RR) 1.05, 95% confidence interval (CI) = 0.78–1.41), with moderate heterogeneity (I^2^ = 36%). Antibiotic treatment was found to present an uncertain risk for the development of drug-resistant strains (RR = 1.51, 95% CI = 0.95–2.40, I^2^ = 0%). In all trials, no significant difference between study arms was demonstrated regarding patient and graft outcomes, such as graft function, graft loss, hospitalization due to UTI, all-cause mortality, or acute rejection. *Conclusions*: The practice of screening and treating kidney transplant patients for asymptomatic bacteriuria does not curtail the incidence of future symptomatic UTIs, increase antimicrobial resistance, or affect graft outcomes. Whether early treatment of ASB after kidney transplantation (<2 months) is beneficial requires more RCTs.

## 1. Introduction

Kidney transplantation(KT) is the best treatment for patients with end-stage renal disease(ESRD). Infection-related complications are one of the leading causes of morbidity and mortality in kidney transplant recipients(KTRs). Due to the use of immunosuppressants and anatomical changes, urinary tract infections (UTIs) constitute the most common complication and a significant source of morbidity among renal transplant recipients [1]. Owing to the different diagnostic criteria for UTI among centers, the incidence rate varies from 6% to 83% [2,3,4]. Acute allograft pyelonephritis represents the severest form of UTIs, often resulting in bacteremia and acute graft dysfunction within this population [5,6]. The majority of UTI complications occur within the initial six months post-transplantation, after which their frequency and severity typically diminish [7]. Enteric Gram-negative bacilli and Enterococcus spp. species are the most common pathogenic bacteria, and their resistance remains a serious problem in the management of UTIs, including among kidney transplant recipients [8]. 

Symptomatic UTIs necessitate comprehensive antibiotic treatment. Although ASB can occur in more than 50% of KTRs in the first year after their KT, the potential benefits of routine screening and treatment of asymptomatic bacteriuria (ASB) among KTRs remain a topic of debate [9,10,11]. This is because of uncertainty about the impact on overall prognosis in transplant patients and the risk of developing symptomatic urinary tract infections and worse graft outcomes. Given this uncertainty, most physicians currently prescribe systemic antibiotics for such patients. It is reported that ASB is always screened and treated by over 70% of transplant physicians during post-kidney transplant surveillance [12]. Apart from the uncertainty surrounding the risk of symptomatic UTIs, there are concerns that pyelonephritis might remain asymptomatic due to the high doses of immunosuppressants administered during the early post-transplant period and graft denervation [13].

Because of the lack of a uniform standard for the use of antibiotics in such situations, it can favor the development of multidrug-resistant (MDR) bacteria. In addition to the long-term use of antibiotic prophylaxis for preventing Pneumocystis jirovecii pneumonia, fluoroquinolones are the most commonly prescribed drugs for ASB after transplantation, which further promotes the selection of resistant organisms. Infections caused by MDR microorganisms have become a serious problem in KT recipients as they are associated with higher rates of complications and relapse [14]. In addition, MDR infections require antibiotic treatment as a last resort, which often results in more adverse events, such as nephrotoxicity, which may be aggravated by the concomitant use of calcineurin inhibitors [15]. In addition, MDR bacterial infections have become a serious problem across the world and deserve more attention for transplant patients. Given this worrying situation, effective antibiotic stewardship strategies are urgently needed to reduce the rate of drug resistance. It is important for us to recognize the key role of antibiotic pressure as the main driving force of MDR bacterial selection. 

In light of the scarcity of compelling evidence, published guidelines offer little in the way of concrete recommendations regarding the screening and treatment of ASB episodes in kidney transplant recipients. A consensus published by the Spanish Network for Research in Infectious Disease (REIPI) recommended that ASB should be systematically screened for and treated up to 1 month post-transplantation [16]. More recently, the guideline on “Urinary Tract Infections in Solid Organ Transplant Recipients” published by the American Society of Transplantation Infectious Diseases Community of Practice suggested that screening and treatment of ASB in renal transplant recipients who have been transplanted for more than two months are not recommended [17]. However, the studies included in these guidelines were of low quality and had a limited sample size. 

Since current guidelines do not encompass the findings of the most recent randomized controlled trials (RCTs), we embarked on a systematic review and meta-analysis of all available RCTs examining the benefits and risks of ASB treatment with antibiotics in kidney transplant recipients, aiming to prevent symptomatic UTIs, prevent graft function deterioration, and reduce unnecessary antibiotic use.

## 2. Methods

This systematic review and meta-analysis were conducted in accordance with the PRISMA (Preferred Reporting Items for Systematic Reviews and Meta-Analyses) statement and the guidelines of the Cochrane Handbook of Systematic Reviews and Meta-Analyses of Interventions [18,19]. The International Prospective Register of Systematic Reviews (PROSPERO) has assigned the number CRD42023451064 to our systematic review procedure.

### 2.1. Types of Studies, Participants, and Interventions

All RCTs and quasi-RCTs assessing the advantages of treating asymptomatic bacteriuria in kidney transplant recipients were included in our study. Our inclusion criteria regarding patients were as follows: adults and children with end-stage kidney disease who were recipients of a first or subsequent kidney transplant from a deceased or living donor, including combined grafts. Our exclusion criteria regarding patients were as follows: pregnant women, as antibiotic treatment of asymptomatic bacteriuria during pregnancy is effective in reducing the risk of pyelonephritis in the mother and may reduce the chance of the baby being born prematurely or having a low birth weight; and transplant recipients awaiting a urologic procedure during which mucosal bleeding was anticipated, as antibiotic treatment of asymptomatic bacteriuria is recommended in this setting. The definition of asymptomatic bacteriuria adhered to that proposed by the Infectious Diseases Society of America (IDSA). The IDSAs definition of asymptomatic bacteriuria is as follows: in men, a single, clean-catch voided urine specimen with one bacterial species isolated in a quantitative count ≥ 105 CFU/mL in the absence of symptoms or signs of UTI; in women, two consecutive voided urine specimens with isolation of the same bacterial strain in quantitative counts ≥ 105 CFU/mL in the absence of symptoms or signs of UTI; or a single catheterized urine specimen with one bacterial species isolated in a quantitative count ≥ 100 CFU/mL, which identifies bacteriuria in women or men. Studies were included regardless of stent, surgical technique, immunosuppression regimen, or antibiotic prophylaxis regimen. Our inclusion criteria encompassed studies comparing any antibiotic regimen—irrespective of antibiotic type, course, or dosing schedule—with placebo or no treatment in renal transplant patients. 

### 2.2. Types of Outcome Measures

#### 2.2.1. Primary Outcomes

The rate of symptomatic UTIs was defined as the isolation of a bacterial species from patients with signs or symptoms of a UTI. The rate of antimicrobial resistance was defined as the isolation of multidrug-resistant bacteria that have non-susceptibility to at least one agent in three or more antimicrobial categories. 

#### 2.2.2. Secondary Outcomes

At the end of this study follow-up period, the following were assessed: all-cause mortality, graft loss, graft rejection, graft function, hospitalization due to UTI, and relapse or persistent asymptomatic bacteriuria. 

### 2.3. Search Strategy

Two authors independently conducted database searches using PubMed, the Cochrane Central Register of Controlled Trials, Embase, Lilacs, and EBSCOHost. We also searched the references of review articles, relevant studies, and clinical guidelines. Unpublished or incomplete studies were sought through personal contact with the investigators. No language or date restrictions were imposed. The most recent search was conducted on 1 March 2023. We searched using: (kidney transplantation [MeSH terms]) combined with the terms (urinary tract infections OR bacteriuria OR asymptomatic bacteriuria OR leukocyturia OR uti OR utis). The search strategies can be viewed in the Supplementary Materials (Table S1).

### 2.4. Assessment of Risk of Bias in Included Studies

Two authors independently evaluated the risk of bias using a specified risk of bias assessment tool [20]. The items of the risk of bias assessment tool are as follows: (1) Was there adequate sequence generation (selection bias)? (2) Was allocation adequately concealed (selection bias)? (3) Was knowledge of the allocated interventions adequately prevented during the study? (4) Were incomplete outcome data adequately addressed (attrition bias)? (5) Were reports of the study free of suggestions of selective outcome reporting (reporting bias)? (6) Was the study apparently free of other problems that could put it at risk of bias? During the above process, when the two authors had different opinions, they discussed and resolved the issue together. If the problem could not be solved, the third author participated in the discussion and made a decision. 

### 2.5. Data Collection and Analysis

Two independent reviewers performed data extraction utilizing standard data extraction forms. If data about ASB, UTI, or randomization methods were not reported, we contacted the authors to obtain these data. Non-English language studies were translated before the assessment. When a study had more than one publication, we selected the report with the most complete data for inclusion in our study.

The mean difference (MD) was employed for continuous variables, specifically to assess treatment effects on graft function. For dichotomous outcomes (symptomatic UTI and graft loss and rejection), the results were expressed as an absolute risk difference (RD) or risk ratio (RR) with a 95% confidence interval (CI). Heterogeneity was evaluated using a chi-squared test, with an alpha of 0.05 set as indicating statistical significance, and the I^2^ measure applied for an inconsistency test [21]. The data were pooled using a fixed-effects model when I^2^ was <30%; otherwise, a random-effects model was adopted. We planned to perform subgroup analyses, including the duration from transplantation to asymptomatic bacteriuria, presence of a ureteric stent, age, sex, deceased donor, pathogens, dose and duration of the antimicrobial therapy, and antimicrobial agent used, to explore possible sources of heterogeneity. However, the paucity of data precluded any subgroup analysis. The analysis was conducted utilizing RevMan 5.3 software (Review Manager [RevMan], Version 5.3, Copenhagen, Denmark: The Nordic Cochrane Centre, The Cochrane Collaboration, 2014). 

## 3. Results

### 3.1. Description of Studies

**Figure 1:**
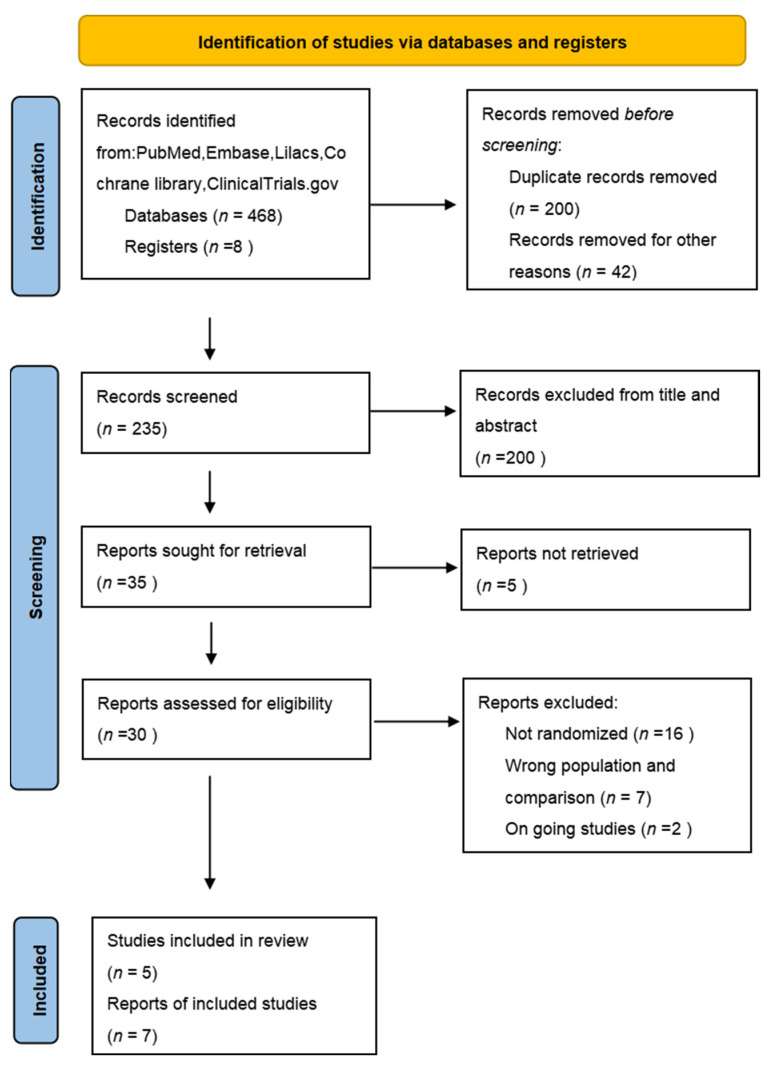
Flow diagram of included and excluded studies.

**Figure 2:**
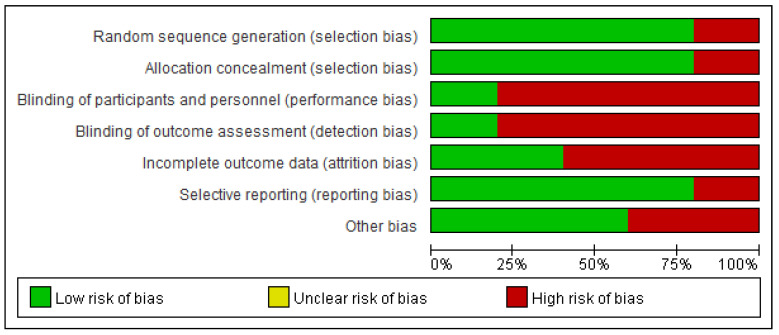
Risk of bias graph: review authors’ judgments about each risk of bias item presented as percentages across all included studies.

**Figure 3:**
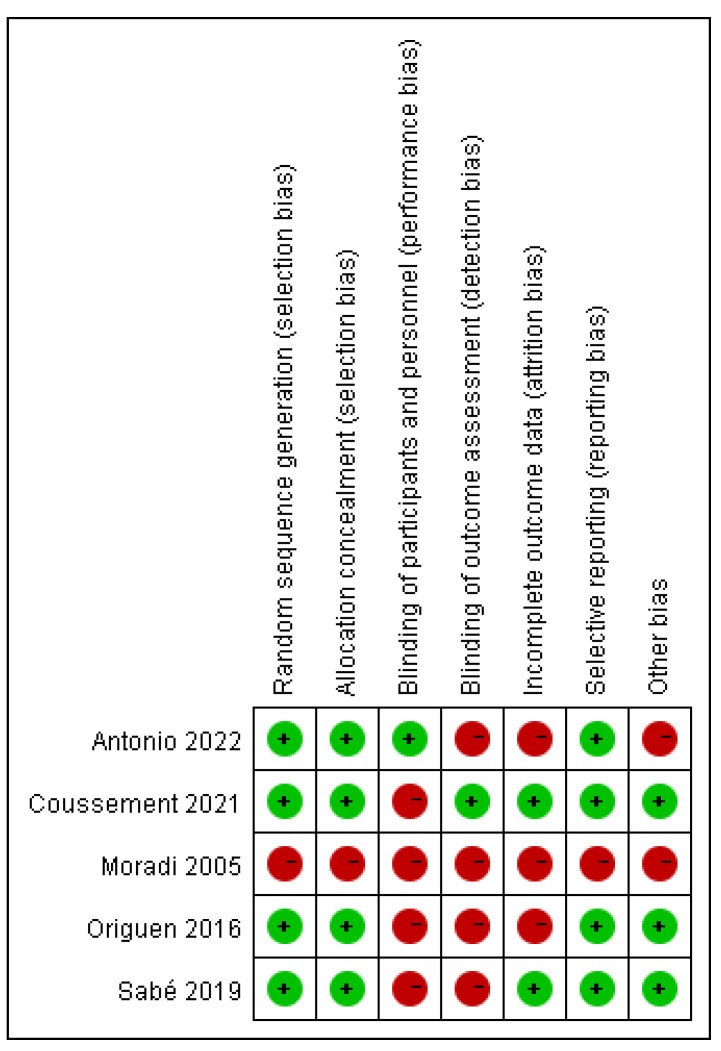
Risk of bias summary: review authors’ judgments about each risk of bias item for each included study.

In total, our electronic searches yielded five studies [22,23,24,25,26] encompassing 566 patients for the meta-analysis (Figure 1). The characteristics of the included studies are detailed in Table 1. Two ongoing studies were not included because of undisclosed data [27,28]. All included studies compared the effects of antibiotics to no treatment on asymptomatic bacteriuria in kidney transplantation recipients. There were heterogeneities among these studies in terms of antibiotic dose, choice, route of administration, and duration. The incidence of symptomatic UTIs was evaluated in all studies. Four studies assessed the rate of antimicrobial resistance, hospitalization due to UTI, acute rejection, and all-cause mortality. Graft function and loss were evaluated in three studies. The risk of bias in the included studies is presented in Figure 2 and Figure 3. One study was graded as having a high risk of bias on all items [22]. Four studies were classified as having a low risk of bias in terms of random sequence regeneration, allocation concealment, and selective reporting [22,23,24,25]. Only one study had a low risk of bias regarding the blinding of participants and personnel [26]. Moreover, only one study had a low risk of bias regarding the blinding of outcome assessment [25]. Three studies were graded as having a high risk of bias due to incomplete outcome data [22,23,26]. 

### 3.2. Primary Outcomes

#### 3.2.1. Symptomatic UTI

**Figure 4:**
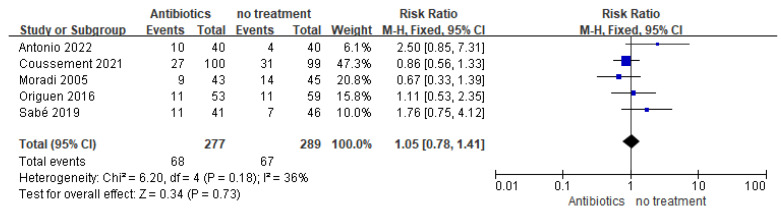
Forest plot of comparison: 1 Antibiotics vs. no treatment; Outcome Symptomatic urinary tract infection.

The outcome of symptomatic UTIs until the end of follow-up was reported in five studies, totaling 566 participants. The incidence of symptomatic UTI varied between 10.0% and 31.3% in the groups not treated for asymptomatic bacteriuria. No significant difference in symptomatic UTI rates was found between antibiotic and no-treatment groups (relative risk (RR) = 1.05, 95% confidence interval (CI) = 0.78–1.41), with moderate heterogeneity (I^2^ = 36%) (Figure 4). The repeated analysis also demonstrated no significant difference in symptomatic UTI rates between the two groups, with low heterogeneity (four studies, 478 participants, RR = 1.18, 95% CI = 0.77–1.80, I^2^ = 28%), after one outlier study [22] was excluded. Four studies [23,24,25,26] reported the rate of graft pyelonephritis and found no difference between patients who were treated for asymptomatic bacteriuria and those who were not (478 participants, RR = 1.31, 95% CI = 0.75–2.29, I^2^ = 0%). Two studies reported the incidence of bloodstream infection due to UTI, and no significant difference was demonstrated between the two groups (286 participants, RR = 0.55, 95% CI = 0.18–1.70, I^2^ = 0%) [24,25]. Moradi (2005) did not provide a specific definition of UTI, and the other four studies provided a definition of UTI based on a positive urine culture of at least 105 colony-forming units per ml. 

#### 3.2.2. Antimicrobial resistance

**Figure 5:**
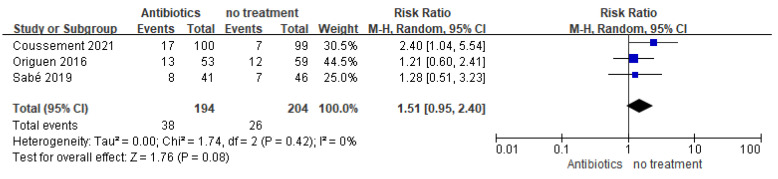
Forest plot of comparison 2: Antibiotics vs. no treatment, outcome: Antimicrobial resistance.

No significant difference in antimicrobial resistance was found between the antibiotic and no-treatment groups (398 participants, RR = 1.51, 95% CI = 0.95–2.40, I^2^ = 0%), even though, numerically, more people in the treatment group had multidrug-resistant bacteria compared to the group not treated for asymptomatic bacteriuria (38/194 vs. 26/204) (Figure 5). Three studies reported the incidence of antimicrobial resistance, which was defined as the number of study participants in whom bacteria with acquired non-susceptibility to at least one agent in three or more antimicrobial categories were isolated during follow-up. Antonio (2022) found a higher incidence of extended-spectrum β-lactamase-producing *E. coli* in the no-treatment group compared to the antibiotic group (12/40 vs. 3/40, *p* = 0.07). 

### 3.3. Secondary Outcomes

#### 3.3.1. Graft Function

**Figure 6:**
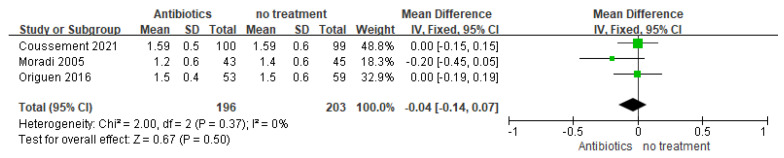
Forest plot of comparison 3: Antibiotics vs. no treatment, outcome: Graft function (creatinine at the end of the study).

Patients in the antibiotic group exhibited similar changes in serum creatinine levels when compared to the no-treatment group (Figure 6, *n* = 399 participants; MD = 0.04 mg/dL, 95% CI = 0.14 to 0.07; I^2^ = 0%). Three studies evaluated the impact of antibiotics on graft function, described as the difference between the creatinine serum levels at the beginning and the end of the study. Moreover, four studies assessed the effect of antibiotics on changes in graft function measured by eGFR from baseline to the end of this study. There was no significant difference between groups in all studies (*p* > 0.05) [22,23,24]. 

#### 3.3.2. Hospitalization due to UTI

**Figure 7:**
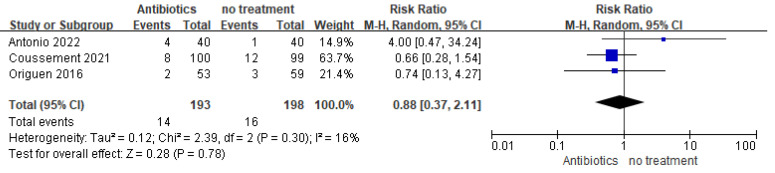
Forest plot of comparison 4: Antibiotics vs. no treatment, outcome: Hospitalization for UTI.

The effect of antibiotics on the rate of hospital admission due to symptomatic UTI was examined in three studies, with the investigators reporting negligible or no difference between the two groups. (Figure 7, 391 participants, RR = 0.88, 95% CI = 0.37–2.11, I^2^ = 16%). In Sabé 2019, hospitalization was defined as any hospital admission due to causes other than acute graft pyelonephritis; thus, the investigators only reported hospitalization due to other reasons such as renal biopsy, pneumonia, and diarrhea. There was still no significant difference when re-comparing the difference in hospitalization rates between the two groups with and without treatment for symptomatic UTIs (RR = 0.75, 95% = CI 0.31–1.83, I^2^ = 0%). 

#### 3.3.3. Persistence or Relapse of Asymptomatic Bacteriuria

Despite the differences not reaching statistical significance, high frequencies of persistent asymptomatic bacteriuria in both groups were noted in three studies. Moradi (2005) reported that bacteriuria recurred in 25 out of 43 treated participants (58.1%) and 33 out of 45 untreated participants (73.3%). The difference was not statistically significant (RD = −0.15, 95% CI = −0.33 to 0.05). Origuen (2016) reported that despite the use of antibiotics, asymptomatic bacteriuria frequently persisted, occurring in 46 out of 131 episodes (35.1%). On the other hand, asymptomatic bacteriuria persisted in 59% (175/296) of untreated episodes and was more common in the control group (RD = −0.24, 95% CI = −0.33 to −0.14). Coussement (2021) also reported a higher incidence of asymptomatic bacteriuria in the no-treatment group at the end of this study (12 months post-study inclusion) compared to the antibiotic group (53% vs. 33%, 49/99 vs. 33/100, *p* = 0.008). However, in Antonio (2022), the rate of recurrent UTI was higher in the antibiotic group compared to the no-treatment group (17.5% vs. 2.5%, 7/40 vs. 1/40, *p* = 0.005). 

#### 3.3.4. Other Outcomes

The outcome of acute rejection up to the end of follow-up was reported in four studies, involving a total of 478 patients. No significant difference between the two groups was demonstrated for this outcome (RR = 0.85, 95% CI = 0.45–1.61), without heterogeneity (I^2^ = 0%) [23,24,25,26]. Three studies (298 patients) reported the outcome of graft loss at the end of follow-up. No significant difference between the two groups was demonstrated for this outcome (RR = 0.77, 95% CI = 0.18–3.36), without heterogeneity (I^2^ = 0%) [23,24,25]. Four studies (478 patients) reported the outcome of all-cause mortality at the end of follow-up. No significant difference between the two groups was demonstrated for this outcome (RR = 1.63, 95% CI = 0.54–4.88), without heterogeneity (I^2^ = 0%) [23,24,25,26]. Additionally, two studies assessed the incidence of diarrhea associated with Clostridioides [23,24]. Origuen (2016 reported) that Clostridioides difficile-associated diarrhea occurred in 3 out of 53 (5.7%) participants from the antibiotic group versus 5 out of 59 (8.5%) participants from the control group. There was no significant difference found between the two groups (RD = −0.03, 95% CI = −0.13 to 0.08).

## 4. Discussion

Based on the five studies, which included 566 patients, antibiotic treatment of asymptomatic bacteriuria in kidney transplant recipients had no effect on preventing subsequent symptomatic UTIs. Although there was no statistically significant difference in antibiotic resistance between the two groups (RR = 1.51, 95% CI = 0.95–2.40, I^2^ = 0%), numerically more people in the treatment group had multidrug-resistant bacteria. Antibiotic treatment presents an uncertain risk for the development of drug-resistant strains, which requires more research to investigate. High frequencies of persistent asymptomatic bacteriuria in both groups were noted, especially in the untreated group; however, differences between the groups did not reach statistical significance. In summary, there is no evidence that antibiotic treatment of asymptomatic bacteriuria in KTRs improves patient and graft outcomes, such as graft function, graft loss, hospitalization due to UTI, all-cause mortality, or acute rejection. 

Although the Infectious Diseases Society of America (IDSA), the American Society of Transplantation, and the European Association of Urology had recommended against the systematic use of antibiotics in kidney transplant recipients with ASB [16,17,29,30], this recommendation was made based on evidence of low certainty. In a recent European survey on the management of asymptomatic bacteriuria among KT recipients, most participants responded that they would treat asymptomatic bacteriuria [12]. Our research further supports the recommendation against systematic antibiotic use in kidney transplant recipients with ASB based on a review of more recent, high-quality clinical studies. However, it is important to be cautious when extrapolating the results of this review to patients in the early stage after kidney transplantation (<2 months), as these patients may receive ureteral stents or indwelling catheters, whose studies were excluded from our review. In the general patient population, those with ureteral stents or catheters are more likely to develop and show persistent asymptomatic bacteriuria due to biofilm formation; however, they do not require treatment. However, in kidney transplant patients with stents or catheters, it is unclear whether screening and treatment for asymptomatic bacteriuria can benefit the patients. Moreover, for patients undergoing kidney combined with other organ transplants, drawing a definitive conclusion needs to be cautious because we currently know very little about the impact of asymptomatic bacteriuria in combined transplant recipients. 

All five studies included in this review involved both male and female adult kidney transplant recipients. Moreover, these studies included kidney transplants from living and deceased donors. Therefore, the applicability of any conclusions to renal transplantation in children is unclear. All studies excluded pregnant patients. Even though screening for and treatment of asymptomatic bacteriuria are considered to effectively reduce the risk of pyelonephritis in mothers and possibly complications in their children [31], our systematic review could not provide any additional information about pregnant women who are kidney transplant recipients.

Traditionally, the detection and antibiotic treatment of asymptomatic bacteriuria are considered strategies to reduce the incidence of complicated UTIs and, thus, enhance patient outcomes and graft survival in kidney transplant recipients. However, the rise of infections caused by multidrug-resistant organisms, including extended-spectrum β-lactamase-producing Enterobacteriaceae and carbapenem-resistant Gram-negative bacilli, poses significant challenges in managing KT recipients [15]. Previous reports only included two studies with low quality and small samples, and the conclusions drawn cannot be further generalized [32]. We included more recent RCTs and found that the administration of antibiotics to kidney transplant patients with asymptomatic bacteriuria did not reduce the incidence of subsequent symptomatic UTIs and pyelonephritis based on the five included RCTs. At the same time, antibiotic treatment poses an uncertain risk for the development of drug-resistant strains, although the combined data of the three included studies did not show a statistically significant difference between groups (RR = 1.51, 95% CI = 0.95–2.40). Persistently high rates of asymptomatic bacteriuria remain high in kidney transplant patients, regardless of treatment. Although urinary tract infections are a risk factor for graft dysfunction and loss, our study found that antibiotic treatment of asymptomatic bacteriuria had no effect on graft loss. Only two studies reported graft loss during the study period; however, this was not related to urinary tract infections or antibiotic treatment. In all trials, no significant difference between study arms was demonstrated regarding patient and graft outcomes such as graft function, hospitalization due to UTI, all-cause mortality, or acute rejection. 

Two studies examined the effect of treating asymptomatic bacteriuria in the early post-transplant period before ureteral stent removal. The results were conflicting. Sabé et al. (2019) reported no differences in symptomatic UTI (12.2%, 5/41 in the treatment group vs. 8.7%, 4/46 in the no-treatment group; RR = 1.40; 95% CI = 0.40–4.87), whereas Antonio (2022) reported dramatic results showing that the incidences of UTI (25% vs. 10%, *p* = 0.07) and pyelonephritis (15% vs. 2.5%, *p* = 0.04) were greater in the intervention group [24,26]. Both studies had a small sample size and different follow-up periods. The types, dosages, and duration of antibiotic use in these two studies were also not consistent. Therefore, more studies are needed to assess whether antibiotic treatment for asymptomatic bacteriuria occurring early after KT can be useful or not in preventing complicated UTIs. 

Our meta-analysis has several limitations, including a high degree of heterogeneity among the included studies. First, the number of studies was small, and there was a lack of well-designed randomized trials evaluating the efficacy of antibiotic therapy in this population. Although four studies reported sample size calculations, only Coussement (2021) reached a relatively large sample size (199 patients) [22,23,24,25,26]. The other four studies had sample sizes ranging from 80 to 112. The samples assigned to each group were even smaller. Thus, a single event could have a large impact on the incidence rate, making it difficult to draw definitive conclusions. The expected incidence of pyelonephritis was overestimated during study planning in two studies, and the planned study sample size was not achieved [23,24]. Second, most of the included studies did not attempt to blind participants, personnel, or data analysts. Since symptoms of UTI are somewhat subjective, we expected this to present a risk of biasing the results toward antibiotic therapy, especially in early postoperative patients. In addition, there was inconsistency regarding how to determine the persistence of asymptomatic bacteriuria during the study period among the different studies. Especially in the no-treatment group, there was no unified plan or completion of systematic urine analysis, which could lead to certain biases in the results. Third, the types of antibiotics and the course of treatment used in the antibiotic treatment of asymptomatic bacteriuria were not completely consistent across studies. Four studies reported isolated bacteria and antibiotic use based on culture results [23,24,25,26], with three studies reporting the antibiotic use categories [23,24,25]. Because of the differences in infecting bacteria, antibiotic use is not uniform. The duration of antibiotic use varied from 3 days to 10 days. A short course of treatment might lead to the occurrence of subsequent symptomatic infections, while a long course of treatment might increase bacterial resistance. Therefore, this could also lead to differences in results. Additionally, the duration of follow-up in the included studies varied, which might have influenced the results. The follow-up duration varied from 63 days to 24 months. Antonio (2022) reported a follow-up of only 63 days, which might underestimate the recurrence rate of infection [26], while the other four studies had a follow-up of more than 12 months. These limitations suggest that additional studies are likely to change our confidence in the effect estimates [29]. 

In conclusion, our findings suggest that screening for and treatment of asymptomatic bacteriuria in kidney transplant patients does not reduce the incidence of subsequent symptomatic UTIs or impact graft outcomes. Antibiotic treatment presents an uncertain risk for the development of drug-resistant strains. However, available data are limited by the small number and low quality of studies. Additional RCTs are needed in order to determine whether antibiotic treatment for asymptomatic bacteriuria occurring early after KT can be useful.

## Figures and Tables

**Table 1:** Characteristics of included trials.

Study	Study Type	Location	Course of Antibiotics in Treatment Group	Number of Patients (Antibiotics vs. No Treatment)	Enrollment Time (Post-Transplantation)	Follow-Up Duration	Outcome: Symptomatic Urinary Tract Infection (Antibiotics vs. no Treatment)
Moradi 2005 [22]	quasi-RCT	Iran	10 days	88 (43 vs. 45)	>1 year	12 months	20.9% vs. 30.4%
Origuen 2016 [23]	RCT	Spain	3–7 days	112 (53 vs. 59)	>2 months	24 months	20.8% vs. 18.6%
Sabé 2019 [24]	RCT	Spain	5–7 days	87 (41 vs. 46)	Post-operation after urinary catheter removal	12 months	26.8% vs. 15.2%
Coussement 2021 [25]	RCT	France and Belgium	10 days	199 (100 vs. 99)	>2 months	12 months	27% vs. 31.3%
Antonio 2022 [26]	RCT	México	5 days	80 (40 vs. 40)	Post-operation after urinary catheter removal	63 days	25% vs. 10%

## Data Availability

Data will be made available upon request to the corresponding author.

## Supplementary Materials

The following supporting information can be downloaded at: https://www.mdpi.com/article/10.3390/medicina59091600/s1, Table S1: Search strategies.
