# Supplementary material for: Treatment of Asymptomatic Bacteriuria after Kidney Transplantation: A Systematic Review and Meta-Analysis of Randomized Controlled Trials
Source: Medicina (Kaunas). 2023 Sep 5;59(9):1600. doi: 10.3390/medicina59091600 (PMC10535591; doi:10.3390/medicina59091600)
Supplement: Supplementary file 1 [file medicina-59-01600-s001.zip › medicina-2560056-supplementary.pdf]

Supplementary Materials: Treatment of Asymptomatic Bacteriuria after Kidney Transplantation: A Systematic Review and Meta-Analysis of Randomized Controlled Trials

Table S1. Search strategies

Database	Search terms
Pubmed, EMBASE, Lilacs, Cochrane library	1. Kidney Transplantation/<br>2. Urinary Tract Infections/<br>3. Bacteriuria/<br>4. uti.tw.<br>5.(bacteriuria and (asymptomatic or covert)).tw.<br>6. or/2–5<br>7. and/1,6
